# South Korean study to prevent cognitive impairment and protect brain health through multidomain interventions via face‐to‐face and video communication platforms in mild cognitive impairment (SUPERBRAIN‐MEET): A randomized controlled trial

**DOI:** 10.1002/alz.14517

**Published:** 2025-01-22

**Authors:** So Young Moon, Yoo Kyoung Park, Jee Hyang Jeong, Chang Hyung Hong, Jiwoo Jung, Hae Ri Na, Soo Hyun Cho, Hyun Sook Kim, Hong‐Sun Song, Muncheong Choi, Bon D. Ku, Yeon Sil Moon, Hyun Jeong Han, Yun Jeong Hong, Eun‐Joo Kim, Geon Ha Kim, Ko Woon Kim, Hyemin Jang, Soo Jin Yoon, Hee‐Jin Kim, Seong Hye Choi

**Affiliations:** ^1^ Department of Neurology Ajou University School of Medicine Suwon Republic of Korea; ^2^ Department of Medical Nutrition Graduate School of East‐West Medical Nutrition Kyung Hee University Yongin Republic of Korea; ^3^ Department of Neurology Ewha Womans University College of Medicine Seoul Republic of Korea; ^4^ Department of Psychiatry Ajou University School of Medicine Suwon Republic of Korea; ^5^ Rowan Inc. Seoul Republic of Korea; ^6^ Department of Neurology Bobath Memorial Hospital Seongnam Republic of Korea; ^7^ Department of Neurology Chonnam National University Medical School Gwangju Republic of Korea; ^8^ Department of Neurology CHA Bundang Medical Center CHA University Seongnam Republic of Korea; ^9^ Department of Physical Education Andong National University Andong Republic of Korea; ^10^ Department of Neurology Catholic Kwandong University International St. Mary's Hospital Incheon Republic of Korea; ^11^ Department of Neurology Konkuk University Medical Center Konkuk University School of Medicine Seoul Republic of Korea; ^12^ Ilsan Brain Neurology Clinic Goyang Republic of Korea; ^13^ Department of Neurology Uijeongbu St. Mary's Hospital The Catholic University of Korea Uijeongbu Republic of Korea; ^14^ Department of Neurology Pusan National University Hospital Pusan National University School of Medicine and Medical Research Institute Busan Republic of Korea; ^15^ Department of Neurology Jeonbuk National University Medical School Jeonju Republic of Korea; ^16^ Department of Neurology Seoul National University Hospital Seoul Republic of Korea; ^17^ Department of Neurology Eulji University Hospital Eulji University School of Medicine Daejeon Republic of Korea; ^18^ Department of Neurology Hanyang University College of Medicine Seoul Republic of Korea; ^19^ Department of Neurology Inha University College of Medicine Incheon Republic of Korea

**Keywords:** cognition, dementia, exercise, lifestyle, mild cognitive impairment, prevention, randomized controlled trial

## Abstract

**INTRODUCTION:**

We investigated the efficacy of a multidomain intervention (MI) via face‐to‐face and video communication platforms using a tablet personal computer application in patients with mild cognitive impairment (MCI).

**METHODS:**

Three hundred participants with MCI and ≥ 1 modifiable dementia risk factor, aged 60‐85 years, were randomly assigned to either the MI group, who underwent a 24‐week intervention, or the control group, who received usual care.

**RESULTS:**

The overall adherence rate to MI was 84.7%. The adjusted mean change from baseline at 24 weeks in the total scale index score of the repeatable battery for the assessment of neuropsychological status was 8.43 in the MI group and 4.26 in the control group (difference, 4.17; 95% confidence interval, 1.92‐6.43; *p* < 0.001). MI showed significant beneficial effects on cognition in both apolipoprotein E (APOE) ε4 carriers and noncarriers.

**DISCUSSION:**

MI can exert beneficial effects on the cognition of patients with MCI.

**Trial Registration**: ClinicalTrials.gov identifier: NCT05023057

**Highlights:**

- Although the controls also demonstrated improved performance in cognition, multidomain interventions showed significantly greater benefits for cognition in MCI compared to the controls in a randomized controlled trial.
- Multidomain interventions improved depression and quality of life.
- Multidomain interventions significantly positively impacted cognition in both APOE ε4 carriers and noncarriers.
- Multidomain interventions may be more effective for amnestic than nonamnestic MCI.

## 1 BACKGROUND

As the global population continues to age, the prevalence of both dementia and mild cognitive impairment (MCI) are rapidly increasing. A recent nationwide population‐based prospective cohort study conducted in Korea estimated the prevalence of MCI in residents aged 60 years or older to be 27%.^1^, ^2^ Although not all MCI cases progress to dementia, approximately 15% of MCI cases progress to dementia within 1 year.^3^, ^4^ As such, preventing this disease progression is needed urgently. Recently, lecanemab and donanemab have been developed as treatments for early Alzheimer's disease (AD), including patients in the MCI stage.^5^, ^6^ Nevertheless, these drugs do not completely prevent the progression of AD, are expensive, and are associated with serious side effects, such as infusion‐related reactions and amyloid‐related imaging abnormalities. Moreover, no drugs are currently available for most cases of MCI due to causes other than AD. Furthermore, according to several neuropathological studies, < 30% of dementia cases exhibit pure AD pathology, with vascular pathology being the most common copathology associated with AD.^7^ Therefore, the identification of nonpharmacological treatments that simultaneously address multiple modifiable risk factors for dementia remains important in the field of MCI research.

Recent studies have shown that lifestyle modifications may prevent dementia in at‐risk individuals.^8^, ^9^, ^10^ The Finnish Geriatric Intervention Study to Prevent Cognitive Impairment and Disability (FINGER) was the first large randomized controlled trial (RCT) involving older individuals with an increased risk of dementia. This trial included dietary counseling, physical exercise, cognitive training, and vascular and metabolic risk monitoring, reporting beneficial effects of intervention on cognition over a 2‐year period.^11^ While the French Multidomain Alzheimer Preventive Trial^12^ and the Dutch Prevention of Dementia by Intensive Vascular Care trials^13^ found no primary outcome effects, exploratory subgroup analyses in both of these trials revealed cognitive benefits in certain at‐risk populations.^13^, ^14^ Additionally, the SoUth Korean study to PrEvent cognitive impaiRment and protect BRAIN health through lifestyle intervention in at‐risk elderly people (SUPERBRAIN) trial demonstrated that adapting the multidomain intervention (MI) outlined in the FINGER study to fit the cultural and circumstantial contexts of another country is feasible and potentially effective.^15^ However, studies investigating the effectiveness of MI targeting only MCI are lacking, as most MI studies enrolled both older adults with MCI and normal cognitive function.^11^, ^12^, ^13^, ^14^, ^15^ A recent small trial showed that a multidomain lifestyle intervention with nutritional supplements was effective in early symptomatic AD.^16^ However, the sample size of this study was small, and the effectiveness of MI alone was not evaluated.

The previous SUPERBRAIN feasibility trial showcased the effectiveness of a tablet‐based MI for older adults, enabling remote adherence monitoring.^15^, ^17^ In the study, both facility‐based MI (FMI) and home‐based MI (HMI) were found to exhibit significant adherence, achieving cognitive improvements compared to controls.^15^ Exploratory analyses highlighted notable brain derived neurotrophic factor‐related benefits in the FMI group.^15^, ^18^ Moreover, the FMI participants displayed an increased mean global cortical thickness, notably in the bilateral frontotemporal lobes, cingulate gyri, and insula, indicating potential structural neuroplastic changes facilitating learning and neurotrophic factors.^18^ While both interventions share content similarities, the intense social interaction aspect of the FMI is noteworthy. Considering factors such as pandemics and environmental concerns, integrating facility‐based elements into home‐based interventions through video communication platforms may allow effective simulation of group dynamics.

**RESEARCH IN CONTEXT**

1. **Systematic review**: The PubMed database was searched to review the literature on the effects of multidomain lifestyle interventions on cognitive functioning in elderly people. Studies investigating the effects of multidomain interventions, specifically in individuals with mild cognitive impairment (MCI), are rare, and none have yet shown meaningful results.
2. **Interpretation**: This study suggests that multidomain interventions have significant beneficial effects on cognition in individuals with MCI. The multidomain interventions positively influenced depression, quality of life, physical performance, dietary habits, and motivation. Additionally, subgroup analyses showed that multidomain interventions exerted significant beneficial effects on cognition in both apolipoprotein E (APOE) ε4 carriers and noncarriers, and that multidomain interventions seemed to be more effective in amnestic than nonamnestic MCI.
3. **Future directions**: Further studies are required to evaluate the efficacy of programs tailored based on the causes of MCI and the efficacy of combined treatment with multidomain interventions and lecanemab or donanemab.

In the present study, we aimed to investigate the efficacy of MI through both face‐to‐face and video communication platforms using a tablet personal computer (PC) application in patients with MCI, compared to a control group receiving usual care.

## 2 METHODS

### 2.1 Study design and participants

The rationale and design details of this study have previously been published.^19^ In brief, this study comprised a 24‐week, multicenter, outcome assessor‐blinded RCT with a two‐parallel‐group design, conducted at 17 hospitals across South Korea. The MI group was set as the experimental group, and the waitlist control group was set as the comparator group. Participants were older adults who visited outpatient clinics of each study center for cognitive decline. Eligible participants were recruited after applying the following inclusion criteria: age of 60‐85 years; at least one modifiable dementia risk factor, such as hypertension,^20^ diabetes mellitus,^21^ dyslipidemia,^22^ obesity,^23^ abdominal obesity,^24^ metabolic syndrome,^25^ smoking,^26^ educational level of ≤ 9 years, social isolation,^27^ and physical inactivity^28^; complaints of cognitive decline by the participant themselves or an informant; a performance score lower than 1.0 standard deviation (SD) below the age‐ and education‐adjusted normative means for one or more of the delayed recall, naming, visuoconstruction, attention, and executive function tests; a Mini‐Mental State Examination (MMSE) Z score ≥ −1.5, based on normative data for Korean adults from a previous study^29^; ability to perform independent activities of daily livings (ADL); ability to use the tablet PC either following education or with personal help; and having a reliable informant who can provide investigators with the requested information. The exclusion criteria were as follows: major psychiatric illness; dementia; other neurodegenerative diseases; history of malignancy within the last 5 years; cardiac stent or revascularization within 1 year; serious or unstable symptomatic cardiovascular diseases; other serious or unstable medical diseases; severe loss of vision, hearing, or communicative disability; illiteracy; any conditions preventing cooperation with the interventions, as determined by a study doctor; any significant laboratory abnormality that may have resulted in cognitive impairment; inability to safely participate in the exercise program; and simultaneous participation in any other intervention trial. Study doctors at each center assessed and ensured inclusion and exclusion criteria for participants.

This study was conducted in accordance with the International Conference on Harmonization Good Clinical Practice Guidelines. The institutional review board of each hospital approved the study protocol, and all consent forms before the study was initiated. Written informed consent was obtained from all participants prior to enrollment. This trial was registered with ClinicalTrials.gov (NCT05023057).

### 2.2 Randomization

Participants were randomly assigned to either the MI or control group in a 1:1 ratio at baseline. The participants were randomized using a permuted block randomization method, with block sizes of two and four, through SAS version 9.4 (SAS Institute Inc., Cary, NC, USA), and were stratified by the participating center. The allocation sequence was only known to the independent statistical specialist. The cognitive outcome assessors remained blinded to treatment allocation. The participants were instructed not to discuss their study involvement with the outcome assessor.

### 2.3 Intervention

Participants in the MI group received the following five intervention components ^19^ : monitoring and management of metabolic and vascular risk factors, cognitive training, physical exercise, nutritional education, and motivational enhancement. The intervention period lasted for 24 weeks. Participants in the MI group were provided with a tablet PC to perform the intervention using the SUPERBRAIN application and ZOOM. They were further provided with elastic bands and nine‐floor plates to facilitate the performance of physical exercise at home. Each group comprised fewer than eight persons, depending on the size of the study center. Participants in the MI group visited a facility once every 1–2 weeks to participate in face‐to‐face interventions in the group or individual sessions, and performed other interventions using the SUPERBRAIN application and ZOOM platform at home. During the first face‐to‐face intervention, we provided a guidebook and training on how to use a tablet PC and access ZOOM, as well as additional training during each subsequent visit, as necessary.

Six individual sessions involving monitoring and management of metabolic and vascular risk factors, 12 group sessions to provide nutritional education, and four group sessions to provide motivational enhancement were conducted through face‐to‐face interventions at a facility. Cognitive training was conducted using the tablet PC SUPERBRAIN application on a tablet PC. Cognitive training targets the cognitive domains of episodic memory, executive function, attention, working memory, calculation, and visuospatial function. The detailed structure and content of the cognitive training application have been previously reported.^17^ Participants underwent group cognitive training sessions (lasting 50 min), led by qualified health professionals, such as psychologists, occupational therapists, or study nurses once every 1–2 weeks at one of the designated facilities. Additionally, they engaged in one or two weekly self‐administered cognitive training sessions at home lasting 30–40 min. Participants also engaged in online cognitive training sessions once or twice weekly, focused on homework and facilitated by a qualified health professional via the ZOOM platform. The physical exercise program included aerobic exercises, exercises to improve balance, activities to enhance flexibility, muscle‐strengthening exercises targeting major muscle groups, and movements involving the fingers and toes. Exercise sessions were conducted thrice per week, each lasting 50 min. Qualified exercise professionals led exercise programs face‐to‐face at the facility, and also conducted exercise programs at home via the ZOOM platform. The exercise intensity was elevated every eight weeks, and the content of the exercise was modified.^19^ Participants attended group exercise sessions led by a qualified exercise professional once every 1–2 weeks at a facility. They participated in two or three weekly online exercise sessions led by a qualified exercise professional at home via the ZOOM platform. Detailed interventions are described in Table S1.

At baseline, participants in the control group met a study doctor, were prescribed medication when necessary, and received educational booklets corresponding to their risk factors and a booklet on lifestyle guidelines to prevent dementia. They further received standard care for cognitive impairment and vascular risk factors during the study period and were informed that they could participate in the MI program after the study had ended.

Antidepressants, anxiolytics, or acetylcholinesterase inhibitors, consumed in stable doses for more than 8 weeks prior to the baseline, were continued without dose changes until the end of the study.

### 2.4 Outcomes

The primary outcome was the change in the total scale index score (range, 40–160) of the Repeatable Battery for the Assessment of Neuropsychological Status (RBANS) with the normative data on Korean adults through a previous study,^30^ from baseline to study completion. Changes in index scores (range, 40–160) of each cognitive domain of the RBANS from baseline to study completion were included as secondary outcomes. The secondary outcomes also included changes in cognition, mood, disability, quality of life (QOL), physical fitness, nutrition, vascular risk factors, motivation, and sleep, as well as conversion to dementia. Cognition was also assessed using MMSE (range, 0–30),^29^ Clinical Dementia Rating Scale‐Sum of Boxes (CDR‐SB) (range, 0–18),^31^ and Prospective Retrospective Memory Questionnaire (PRMQ) (range, 16–80) to assess proxy‐ and self‐perceived memory problems.^32^ Mood was assessed using the Geriatric Depression Scale‐15 items (GDS‐15) (range, 0–15),^33^ disability was evaluated using the Bayer ADL (range, 1–10),^34^ and QOL was assessed with the QOL‐AD (range, 0–52).^35^ Physical fitness was evaluated using the Global Physical Activity Questionnaire,^36^ Short Physical Performance Battery (SPPB) (range, 0–12),^37^ sit‐to‐stand for 30 s, and 2‐min stepping test. Nutrition was assessed using the Mini Nutritional Assessment (range, 0–14),^38^ and Nutritional Quotient for Elderly (NQ‐E) (range, 0–100).^39^ The NQ‐E was developed by the Korean Nutritional Society to assess how often participants eat vegetables, fruits, beans, fish, milk, dairy products, eggs, water, fast food, pastries, and sweet foods. Motivation was assessed using the Self‐Determination Index (SDI).^19^ Vascular risk factors were evaluated by assessment of blood pressure, body mass index, waist circumference, smoking, alcohol consumption, lipid profile, hemoglobin A1c, and fasting glucose level. Sleep was evaluated using the Pittsburg Sleep Questionnaire Index (PSQI) (range, 0–21).^40^ We further investigated the progression from MCI to dementia among participants. The diagnosis of dementia was based on the Diagnostic and Statistical Manual of Mental Disorders, Fifth Edition, Text Revision criteria (DSM‐V‐TR) for dementia.^41^ The diagnosis of AD dementia (ADD) was based on the core clinical criteria for probable ADD established by the National Institute on Aging‐Alzheimer's Association workgroups.^42^ Higher scores on the RBANS, MMSE, QOL‐AD, SPPB, 30‐s sit‐to‐stand test, 2‐min stepping test, MNI, NQ‐E, and SDI indicate better performance. Lower CDR‐SB, GDS‐15, Bayer ADL, PRMQ, and PSQI scores further suggest better performance.

Whether conducted at a facility, or through the ZOOM platform or telephone, participant adherence to both group and individual interventions was evaluated based on real‐time attendance at the intervention sessions. The tablet‐based cognitive application was further configured to allow administrators to view all participants’ data on the administration homepage. Study coordinators evaluated adherence to the self‐administered cognitive training sessions at home via the administration homepage. Tolerance was assessed via assessment of the retention rate, which involved the calculation of the percentage of participants in each group who did not drop out by the end of the study. Study coordinators evaluated the occurrences of adverse events (AEs) when participants visited a facility, when AEs took place, and at the end of the study. The safety committee met regularly to assess any adverse events.

The RBANS was administered by the same psychologist at assessment performed at baseline, week 12, and within 4 weeks of the end of the intervention. Other secondary outcomes were evaluated 4 weeks prior to the intervention and within 4 weeks after the intervention. Participants who withdrew prematurely were asked to complete all endpoint assessments at the point of early termination. All primary and secondary outcome measures were assessed face‐to‐face at each study center.

### 2.5 Statistical analysis

In our previous study, the difference in the change in RBANS total scale index score between the control and HMI groups was 6.2.^15^ The SD of the RBANS total scale index score was 19.8 in the control group, among whom the autocorrelation of the RBANS total scale index scores between baseline and study end was 0.8. In this trial, the RBANS was assessed three times. To achieve a power of 0.8 for detecting a significant difference (*p *= 0.05, two‐sided) using the time‐averaged difference of repeated measures in the power analysis and sample size program PASS 11 (NCSS, Kaysville, UT, USA), 134 participants in each group were required. Accounting for an anticipated dropout rate of 10.5%, based on our previous findings,^15^ we estimated a total required sample size of 300 participants, with 150 participants in each group.

The adherence rate was calculated by adding the number of sessions completed and dividing it by the number of interventions assigned to each intervention component: vascular and metabolic risk factor management (6 sessions), cognitive training (48 sessions), physical exercise (72 sessions), nutrition education (12 sessions), and motivational enhancement (4 education sessions and 24 self‐assessments of dementia prevention activities). The total adherence rate was subsequently calculated by summing the number of sessions completed across all intervention components, and dividing this number by the total number of interventions (166 sessions) assigned without modifying the weight of each intervention component. The chi‐squared test was applied to compare retention rates between the intervention and control groups.

Efficacy analyses were performed in the modified intention‐to‐treat (mITT) population, defined as a group of randomly assigned participants who participated in the intervention program at least once and had a baseline assessment and at least one postbaseline primary efficacy RBANS measurement. Additional analyses were also conducted on the per‐protocol population, defined as all participants who completed the study without major protocol deviations. Safety was evaluated in the safety population, defined as the group of participants who underwent at least one safety evaluation at baseline, and participated at least once in the intervention program.

We applied the chi‐squared test for categorical variables and Student's *t*‐test for continuous variables to compare baseline characteristics between the groups. Primary analysis of the change in the RBANS total scale index score from baseline to 24 weeks was further performed to compare the MI and control groups, using a mixed model for repeated measures that included subject and study center as random effects and the baseline RBANS total scale index score as a covariate, with trial group, visit, center by recruitment rate (≥ 2 subjects per month or < 2 subjects per month), and[Fig alz14517-fig-0001] trial group‐by‐visit interactions as the fixed effects. The skewness and kurtosis of the residuals converged to 0, showing an approximately normal distribution. Imputation for missing values was not performed in the primary analysis as the rate of missing values was relatively low, around 10%. Further, no significant differences in baseline characteristics were observed between the participants (*n* = 28) with one postbaseline primary outcome assessment and those (*n* = 249) with two assessments after baseline, indicating minimal risk of bias due to missing values. Additionally, sensitivity analyses were conducted to verify the robustness of the primary outcome analysis results. Multiple imputation analysis was performed, as the data were deemed to be missing at random. The imputation model included group, age, sex, education, APOE ε4 status, and baseline RBANS total scale index score as predictors. Missing data were imputed using the fully conditional specification method with regression analysis, generating 10 imputed datasets. After analyzing each dataset, Rubin's pooling method was applied to combine the results. The change from baseline to 24 weeks in each cognitive domain index score of the RBANS was compared between the MI and control groups, using a mixed model for repeated measures that included subject and study center as random effects and the baseline cognitive domain index score as a covariate, with trial group, visit, center by recruitment rate (≥ 2 subjects per month or < 2 subjects per month), and trial group–by–visit interactions as fixed effects. To compare changes from baseline to the study endpoint in other secondary outcomes between the MI and control groups, an analysis of covariance (ANCOVA) with the baseline score as a covariate was performed if changes in each group's outcomes were normally distributed. When changes were not normally distributed, a nonparametric partial correlation analysis, adjusted for the baseline score, was conducted to assess the relationship between treatment group and outcome change. The Cox proportional hazards model was further applied to examine the effects of MI on dementia progression. Data are presented as the hazard ratios (HR) and 95% confidence interval (CI). Survival was defined as the time between entering the study and the progression of dementia or censoring events, such as withdrawal from the study and the last completed follow‐up examination. The event was classified as a progression to dementia.

Subgroup analyses were performed as specified in the statistical analysis plan. Changes in the RBANS total scale index score from baseline to the end of the study were compared between the MI and control groups subdivided by sex (male or female), APOE ε4 carrier status, or amnestic MCI and nonamnestic MCI for subgroup analyses, using a mixed model for repeated measures that included subject and study center as random effects and the baseline RBANS total scale index score as a covariate, and the trial group, visit, center by recruitment rate (≥ 2 subjects per month or < 2 subjects per month), and trial group‐by‐visit interaction as fixed effects. The MMSE score change from baseline to study completion was finally compared between the MI and control groups using an ANCOVA with a baseline MMSE score as a covariate for subgroup analyses. Statistical analyses were performed using SPSS software (version 26.0; SPSS, Chicago, IL, USA). *p* < 0.05 was considered significant.

## 3 RESULTS

### 3.1 Trial population and baseline characteristics

**FIGURE 1 alz14517-fig-0001:**
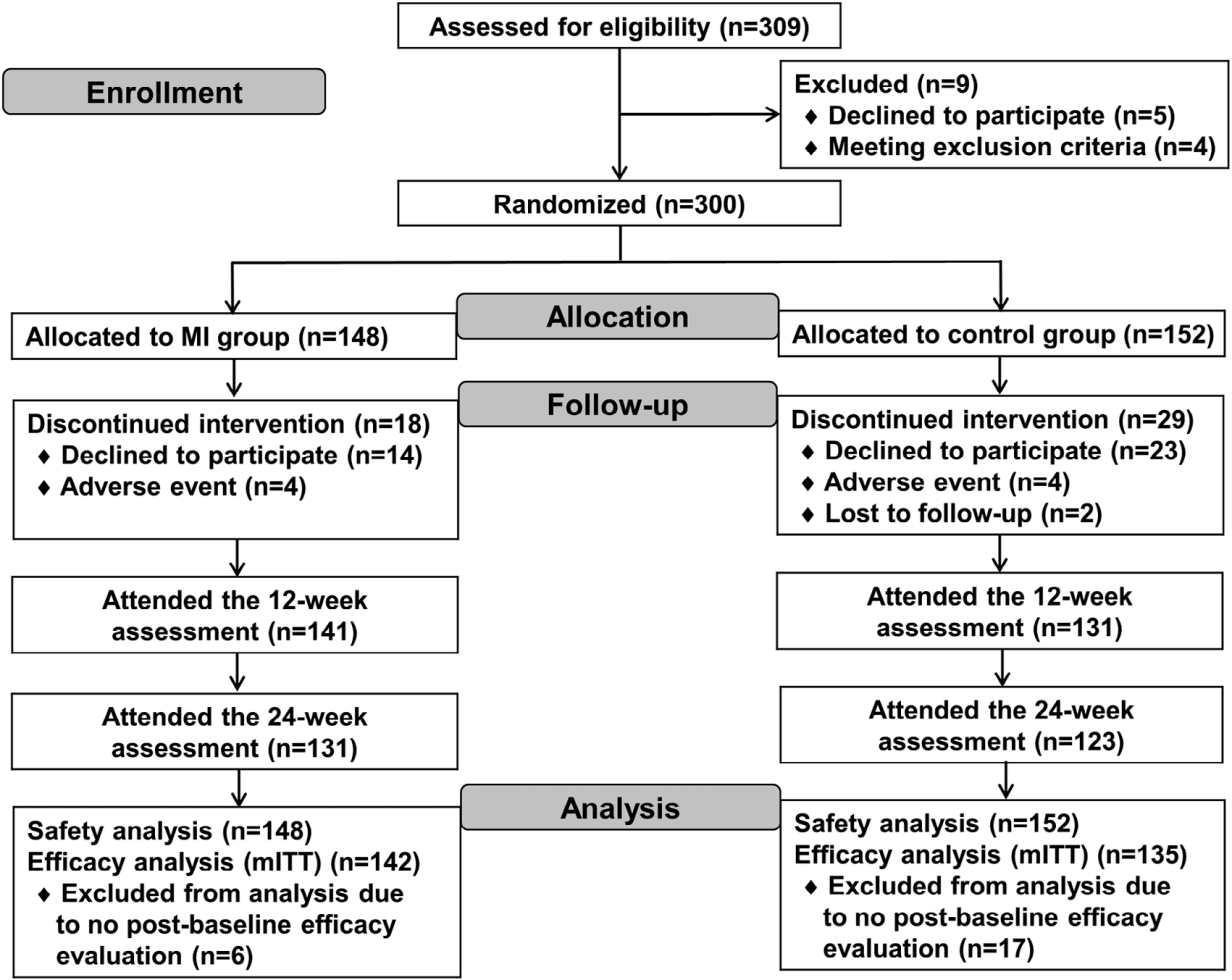
Overview of the trial profile. MI, multidomain intervention; mITT, modified intention‐to‐treat.

Between August 2021 and June 2022, 309 participants were screened for eligibility, of whom 300 underwent randomization; 148 and 152 were assigned to the MI and control groups, respectively (Figure 1). Prescreening was used to reduce screening failures. Five participants withdrew consent and were excluded during screening, while four participants were excluded due to exclusion criteria (two had dementia, one had a positive syphilis serology test, and one lacked a study partner). Recruitment rates at each study center ranged from 0.4 to 5.0 subjects per month. Six study centers had recruitment rates of more than two subjects per month. Study centers that had a referral center for recruitment tended to have higher recruitment rates. Of these participants, 130 (87.8%) in the MI group and 123 (80.9%) in the control group completed the trial, yielding no significant difference in retention rates between the groups (*p *= 0.10). There were no significant differences in age, sex, education, APOE Ɛ4 carriers, GDS‐15 score, and CDR‐SB score at baseline between the participants who discontinued the study and those who completed it. The mITT population included 277 participants (142 in the MI group and 135 in the control group), while the safety population included 300 randomly assigned participants. The baseline demographic and clinical characteristics of the 277 participants included in the mITT analysis are summarized in Table 1. No significant differences were observed in demographic or clinical characteristics between the MI and control groups. In addition, there were no significant differences in age, sex, education, APOE Ɛ4 carriers, treatment group, GDS‐15 score, CDR‐SB score, and RBANS total scale index score at baseline between the participants (*n* = 28) with only one postbaseline primary outcome assessment and those (*n* = 249) with two assessments after baseline.

**TABLE 1:** Characteristics of study participants at baseline (modified ITT population).

Parameter	MI group (*n* = 142)	Control group (*n* = 135)
Age at baseline visit, years	73.0 (5.5)	72.8 (5.6)
Number of women	91 (64.1%)	101 (74.8%)
Education, years	10.7 (4.5)	10.0 (4.2)
Hypertension	67 (47.2%)	70 (51.9%)
Diabetes mellitus	38 (26.8%)	30 (22.2%)
Dyslipidemia	94 (66.2%)	85 (63.0%)
Systolic blood pressure, mmHg	126.4 (13.0)	128.2 (13.5)
Diastolic blood pressure, mmHg	70.7 (10.5)	70.5 (9.0)
Fating plasma glucose, mg/dL	113.5 (37.1)	109.3 (34.5)
Hemoglobin A1c	6.0 (0.6)	6.0 (0.6)
Body mass index, kg/m^2^	23.8 (3.0)	23.8 (3.5)
Abdominal circumference, cm	82.9 (8.9)	82.5 (9.4)
Current smokers	2 (1.4%)	6 (4.4%)
At‐risk alcohol drinking^a^	6 (4.2%)	7 (5.2%)
Low physical activity^b^	32 (22.5%)	31 (23.0%)
Apolipoprotein E ɛ4 carrier	48 (33.8%)	39 (28.9%)
MMSE score	26.5 (2.0) (range, 20‐30)	26.4 (2.5) (range, 19–30)
Geriatric Depression Scale‐15 items score	4.5 (3.7)	5.0 (3.9)
Total scale index score of RBANS	90.0 (15.0)	86.8 (18.8)
Immediate memory index score of RBANS	90.0 (12.9)	88.2 (15.9)
Visuoconstruction index score of RBANS	92.3 (15.4)	90.9 (15.1)
Language index score of RBANS	98.7 (12.8)	96.1 (13.6)
Attention index score of RBANS	100.7 (14.1)	97.2 (14.7)
Delayed memory index score of RBANS	81.2 (17.0)	80.4 (20.4)

*Note*: Values are presented as mean (SD) or number (%).

Abbreviations: ITT, intention‐to‐treat; MI, multidomain intervention; MMSE, mini‐mental state examination; RBANS, Repeatable Battery for the Assessment of Neuropsychological Status.

^a^ Four drinks or more per day or > 7 drinks per week.

^b^ < 600 MET × min/week.

### 3.2 Primary outcome

**FIGURE 2 alz14517-fig-0002:**
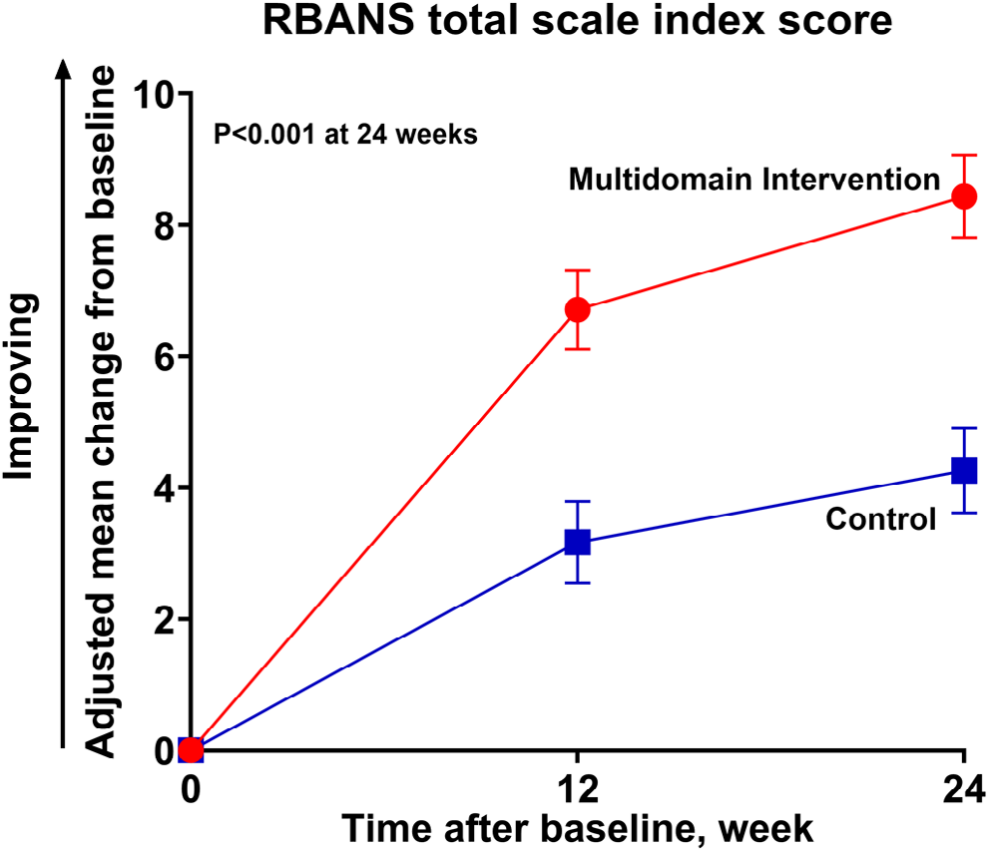
Adjusted mean changes in the RBANS total scale index score from baseline at each visit point. The adjusted mean changes from baseline, standard errors (SE, indicated by bars), and *p*‐value were all derived using a mixed model for repeated measures, with the trial group, visit, center by recruitment rate (≥ 2 subjects/month or < 2 subjects/month), and trial group × visit interaction as fixed effects, subject and study center as random effects, and the baseline value as a covariate. Compared to the control group, the RBANS total scale index score significantly improved in the multidomain intervention group at 12 weeks (6.71 [SE = 0.60] vs. 3.17 [0.62], *p* < 0.001) and 24 weeks (8.43 [0.62] vs. 4.26 [0.64], *p* < 0.001). RBANS, Repeatable Battery for the Assessment of Neuropsychological Status.

As the primary outcome analysis, the adjusted mean changes from baseline at 24 weeks in the RBANS total scale index score were 8.43 (95% CI, 7.21–9.65) and 4.26 (95% CI, 3.00–5.52) in the MI and control groups, respectively (difference, 4.17; 95% CI, 1.92–6.43; *p* < 0.001) (Table 2 and Figure 2). Additionally, the adjusted mean changes from baseline at 12 weeks in the RBANS total scale index score were 6.71 (95% CI, 5.53–7.89) for the MI group and 3.17 (95% CI, 1.95–4.39) for the control group, with a between group difference of 3.54 (95% CI, 1.62–5.46; *p* < 0.001). Cohen's *d* to represent the intervention effect size was 0.43 for the MI group. Sensitivity analyses of the RBANS total scale index score were generally consistent with the primary analysis in the mITT population (Table S2). Additionally, in the per‐protocol population, the RBANS total scale index score in the MI group showed a significant improvement at week 24 compared to the control group (Table S3).

**TABLE 2:** Adjusted mean differences in the change in each index score of the RBANS from baseline to 24 weeks between the multidomain intervention and control groups.

	Adjusted mean change from baseline to 24 weeks (SE)		Adjusted mean between‐group difference in 24‐week change from baseline (95% CI)	
Parameter	MI (*n* = 142)	Control (*n* = 135)	MI versus control	*p*‐Value^a^
**Primary outcome**				
Total scale	8.43 (0.62)	4.26 (0.64)	4.17 (1.92–6.43)	< 0.001
**Secondary outcomes**				
Immediate memory	7.18 (0.68)	5.33 (0.70)	1.85 (−0.68–4.38)	0.151
Visuoconstruction	4.79 (0.80)	−0.39 (0.83)	5.18 (2.14–8.21)	0.001
Language	4.96 (0.78)	2.35 (0.81)	2.62 (−0.30–5.53)	0.079
Attention	1.46 (0.62)	0.31 (0.64)	1.15 (−1.13–3.43)	0.322
Delayed memory	8.66 (0.83)	6.37 (0.86)	2.29 (−0.75–5.33)	0.139

*Note*: ^a^Linear mixed model that included subject and study center as random effects and the baseline score as a covariate, with trial group, visit, center by recruitment rate (≥ 2 subjects per month or < 2 subjects per month), and trial group × visit interaction as fixed effects.

Abbreviations: MI, multidomain intervention; RBANS, Repeatable Battery for the Assessment of Neuropsychological Status.

### 3.3 Secondary outcome

When[Fig alz14517-fig-0002] comparing the change in the index score for each cognitive domain of the RBANS with that of the control group, visuoconstruction ability was significantly improved at 24 weeks in the MI group (Table 2). Compared to the control group, the MMSE score; depression; QOL; dietary habits evaluated by the NQ‐E; physical performance evaluated by the SPPB, 30 s sit‐to‐stand test, and 2 min stepping test; and motivation evaluated by the SDI were significantly improved at 24 weeks in the MI group (Table 3). In addition, the results of the secondary outcome analyses in the per‐protocol population were similar to those in the mITT population (Tables S3 and S4). A total four and nine patients in the MI and control groups developed dementia during the study period, respectively. The HR of conversion to dementia in the MI group was not significantly lower compared with the control group (HR = 0.46; 95% CI, 0.14–1.52; *p *= 0.202). All 13 participants progressed to probable ADD.

**TABLE 3 alz14517-tbl-0003:** Mean changes in the secondary outcome measures from baseline to study end in participants receiving multidomain intervention and controls.

	Baseline scores		Changes from baseline to study end		
Parameter	MI (*n* = 140)	Control (*n* = 123)	MI (*n* = 140)	Control (*n* = 123)	*p*‐Value
Mini‐Mental State Examination^c^	26.5 (2.0)	26.5 (2.5)	0.3 (2.1)	−0.7 (2.4)	<0.001^a^
CDR‐SB	1.13 (0.83)	1.37 (0.91)	−0.04 (0.49)	0.06 (0.64)	0.108^b^
PRMQ	34.5 (10.5)	36.4 (11.3)	−1.7 (9.5)	−1.2 (9.7)	0.212^b^
PRMQ by caregiver	35.7 (10.6)	36.5 (11.8)	−1.2 (9.0)	0.6 (9.8)	0.066^a^
Geriatric depression scale‐15 items	4.4 (3.7)	5.0 (3.9)	−1.3 (3.1)	−0.7 (3.2)	0.010^a^
Bayer ADL	2.24 (1.09)	2.45 (1.30)	−0.004 (1.21)	0.06 (1.32)	0.502^b^
QOL‐AD^c^	33.4 (4.0)	32.2 (5.0)	2.0 (4.3)	0.9 (3.6)	0.003^a^
Pittsburgh sleep quality index	6.2 (4.1)	6.7 (4.0)	−0.3 (3.0)	−0.2 (3.2)	0.373^a^
SPPB^c^	10.1 (1.8)	9.9 (2.0)	0.7 (1.7)	0.2 (1.5)	0.001^a^
30 s sit‐to stand test^c^	13.5 (3.8)	14.0 (4.8)	3.2 (5.1)	0.8 (4.2)	<0.001^b^
2 min stepping test^c^	96.2 (30.4)	96.6 (34.6)	24.7 (30.0)	2.3 (30.7)	<0.001^b^
Nutrition quotient for elderly^c^	67.5 (9.6)	64.4 (10.5)	3.7 (7.1)	0.6 (7.8)	<0.001^a^
Mini nutritional assessment^c^	12.2 (1.9)	11.8 (2.1)	0.1 (1.8)	0.3 (2.2)	0.986^b^
Systolic BP, mmHg	126.1 (12.7)	127.8 (13.5)	0.6 (14.3)	2.0 (15.3)	0.150^a^
Diastolic BP, mmHg	70.6 (10.2)	70.3 (9.0)	−0.3 (10.9)	0.2 (10.8)	0.826^a^
Body Mass Index, kg/m^2^	23.8 (3.0)	24.0 (3.5)	−0.11 (0.81)	−0.01 (1.17)	0.914^b^
HbA1c, %	6.0 (0.6)	5.9 (0.5)	−0.06 (0.30)	−0.04 (0.35)	0.444^b^
Fasting glucose, mg/dL	112.3 (34.3)	106.2 (22.6)	−2.7 (36.9)	−0.7 (28.2)	0.734^b^
Total cholesterol, mg/dL	175.1 (39.1)	187.0 (44.7)	−10.3 (29.5))	−14.9 (41.6)	0.826^b^
LDL‐cholesterol, mg/dL	97.1 (34.2)	105.5 (37.8)	−7.4 (26.4)	−10.9 (35.6)	0.778^b^
HDL‐cholesterol, mg/dL	56.7 (15.4)	57.1 (13.4)	1.0 (8.9)	0.1 (8.7)	0.402^a^
Triglyceride, mg/dL	120.7 (71.5)	140.0 (89.0)	−7.8 (65.5)	−13.5 (84.0)	0.825^b^
SDI^c^	16.5 (19.5)	10.9 (19.8)	9.0 (19.7)	−1.0 (18.0)	<0.001^a^

*Notes*: Values are presented as mean (SD).

Abbreviations: ADL, activities of daily livings; BP, blood pressure; HbA1c, Hemoglobin A1c; CDR‐SB, Clinical Dementia Rating Scale‐Sum of Boxes; HDL, high‐density lipoprotein; LDL, low‐density lipoprotein; MI, multidomain intervention; PRMQ, Prospective Retrospective Memory Questionnaire; QOL‐AD, quality of life in Alzheimer's disease; SD, standard deviation; SDI, Self‐Determination Index; SPPB, Short Physical Performance Battery.

^a^ Analysis of covariance with the baseline score as a covariate.

^b^ Nonparametric partial correlation adjusted for the baseline score between treatment group and change in each outcome from baseline to study end.

^c^ Higher scores indicate better performance.

### 3.4 Adherence

**FIGURE 3 alz14517-fig-0003:**
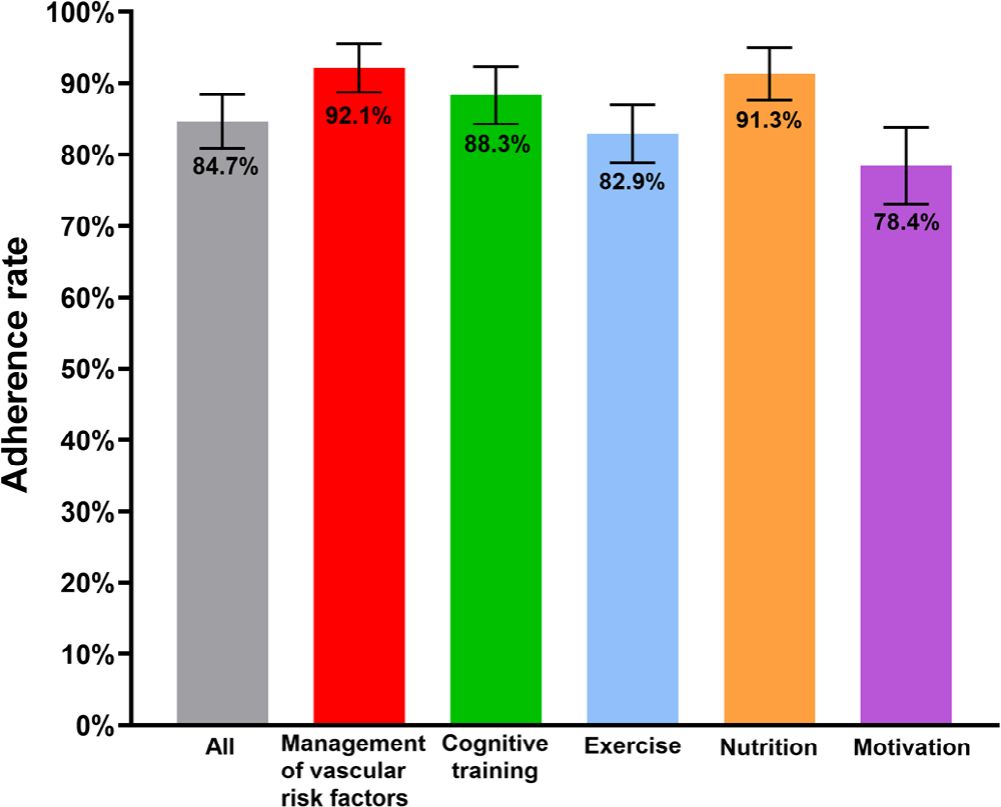
Adherence rates for each intervention domain in the multidomain intervention group. Bars represent the 95% confidence intervals.

The total adherence rate was 84.7% (95% CI, 80.9‐88.4%) in the MI group. The individual adherence rates to the vascular and metabolic risk factor management, cognitive training, physical exercise, nutritional education, and motivational enhancement programs were 92.1% (95% CI, 88.7–95.5%), 88.3% (95% CI, 84.3‐92.3%), 82.9% (95% CI, 78.8‐87.0%), 91.3% (95% CI, 87.6‐95.0%), and 78.4% (95% CI, 73.1‐83.8%), respectively, in the MI group (Figure 3).

### 3.5 Safety

The population for the safety analysis was 300. Table 4 summarizes the AEs that occurred in three or more participants. The incidences of participants reporting at least one AE during the study period were 30.4% and 32.2% in the MI and control groups, respectively (*p* = 0.732). Four intervention‐related AEs (2.7%) were reported in the MI group, including two cases of knee pain and one case each of back pain and rib fracture. There were four cases of fractures as AEs in the MI group, and three participants dropped out; however, all four cases were included in the mITT analysis. Seven serious AEs (SAEs) were reported in each of the MI and control groups (*p *= 0.959). SAEs in the MI group included intracerebral hemorrhage, general weakness, knee ligament rupture, spinal compression fracture, burns, aggravated rheumatoid disease, and pulmonary edema, whereas those in the control group included two cases of cataract surgery, intracerebral hemorrhage, shoulder operation, cerebral infarction, diabetic retinopathy, and Guillain‐Barre syndrome. None of the SAEs was deemed to be related to the study.[Table alz14517-tbl-0003]

**TABLE 4 alz14517-tbl-0004:** Adverse events.

Parameter	MI group	Control group
Any adverse event	45 (30.4%)	49 (32.2%)
COVID‐19 infection	9 (6.1%)	10 (6.6%)
Upper respiratory infection	1 (0.7%)	3 (2.0%)
Musculoskeletal pain	13 (8.8%)	6 (3.9%)
Fracture	4 (2.7%)	0 (0.0%)
Dizziness	1 (0.7%)	4 (2.6%)
Anorexia	0 (0.0%)	3 (2.0%)
Dyspepsia	2 (1.4%)	1 (0.7%)
Diarrhea	1 (0.7%)	2 (1.3%)
Hyperlipidemia	1 (0.7%)	3 (2.0%)
Chest pain	1 (0.7%)	3 (2.0%)
Stroke	1 (0.7%)	2 (1.3%)
Eye surgery	0 (0.0%)	3 (2.0%)
Insomnia	0 (0.0%)	3 (2.0%)

Abbreviations: COVID‐19, coronavirus disease 2019; MI, multidomain intervention.

### 3.6 Subgroup analyses

**FIGURE 4 alz14517-fig-0004:**
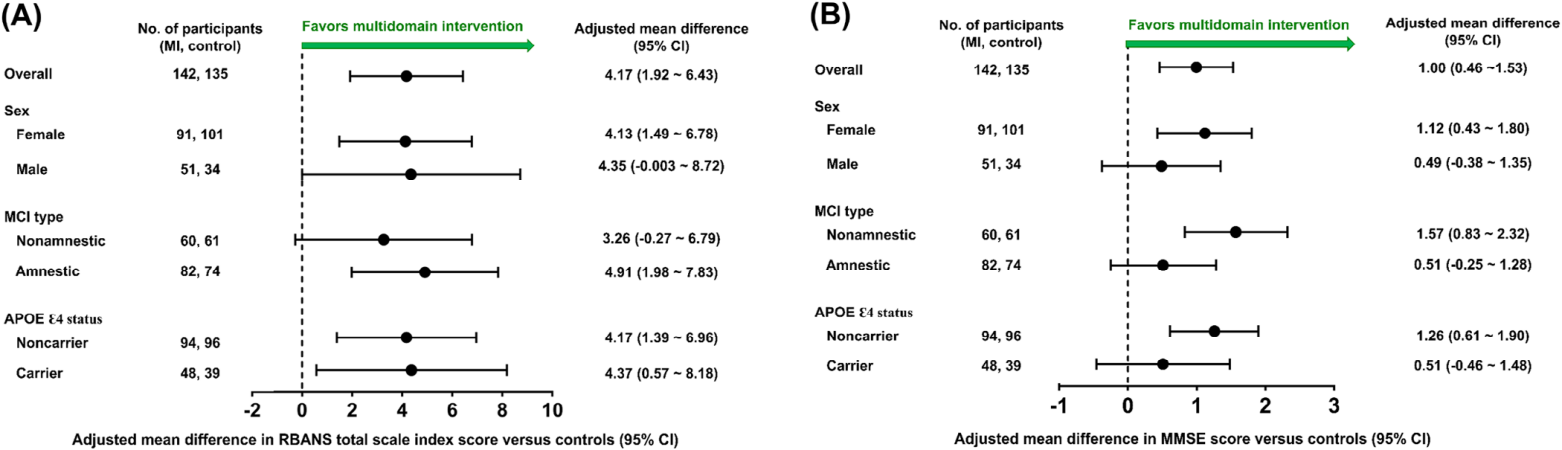
Forest plot of the results of subgroup analysis of the changes in the RBANS total scale index score (A) and MMSE score (B) at 24 weeks by sex, MCI subtype, and APOE genotype. APOE, apolipoprotein E; CI, confidence interval; MCI, mild cognitive impairment; MI, multidomain intervention; MMSE, Mini‐Mental State Examination. RBANS, Repeatable Battery for the Assessment of Neuropsychological Status.

Figure 4 shows the results of the subgroup analysis of the changes in the RBANS total scale index and MMSE scores at 24 weeks according to sex, MCI subtype, and APOE genotype. In females, the RBANS total scale index (difference, 4.13; 95% CI, 1.49–6.78; *p* = 0.002) and MMSE (difference, 1.12; 95% CI, 0.43–1.80; *p* = 0.001) scores showed significant improvements at the study end from baseline in the MI group[Fig alz14517-fig-0003], [Table alz14517-tbl-0004] compared to the control group. Among males, the MI group showed a tendency toward an improvement in the RBANS total scale index score compared to the control group (difference, 4.35; 95% CI, ‐0.003–8.72; *p* = 0.050). In the subanalysis of participants with amnestic MCI, the RBANS total scale index score was significantly improved at the study end from baseline in the MI group compared to the control group (difference, 4.91; 95% CI, 1.98–7.83; *p* = 0.001). In the subanalyses of participants with nonamnestic MCI, the MI group showed a tendency toward an improvement in the RBANS total scale index score (difference, 3.26; 95% CI, ‐0.27–6.79; *p* = 0.070), as well as a significant improvement in MMSE (difference, 1.57; 95% CI, 0.83–2.32; *p* < 0.001) at the study end from baseline compared to controls. In the subanalysis of APOE Ɛ4 carriers, the RBANS total scale index score showed a significant improvement at the study end in the MI group compared to the control group (difference, 4.37; 95% CI, 0.57–8.18; *p* = 0.024). In the subanalyses of APOE Ɛ4 noncarriers, the MI group showed significant improvements in the RBANS total scale index score (difference, 4.17; 95% CI, 1.39–6.96; *p* = 0.003) as well as in the MMSE score (difference, 1.26; 95% CI, 0.61–‐1.90; *p* < 0.001) at the end of the study compared to the control group.

## 4 DISCUSSION

These study showed that MI was effective in improving cognition compared to the control group in patients with MCI. Overall, the RBANS total scale index score improved by an average of 8.43 points in the MI group at the end of the study, which exceeds the minimum clinically important difference of 8 points on the RBANS total scale index score, as outlined in a previous report.^43^ Therefore, the change in the RBANS total scale index score in the MI group could be considered clinically meaningful. The intervention effect of MI as measured by Cohen's *d* was medium. MI was also found to be effective at decreasing depressive symptoms and improving QOL, physical fitness, dietary habits, and motivation among participants with MCI compared to the control group. To our knowledge, this is the first RCT to demonstrate the efficacy of MI targeting MCI alone.[Fig alz14517-fig-0004]

The intervention group demonstrated high adherence to the intervention. In the Japan‐Multimodal Intervention Trial for the Prevention of Dementia, MI appeared to be particularly effective among individuals with high adherence to exercise or cognitive training.^44^ We implemented several strategic efforts to achieve high adherence. First, participants were motivated through group counseling sessions, the Family as a Coach (FAMICO) program, and weekly self‐assessments of their dementia prevention activities.^15^ The FAMICO program involved family members or research staff who encouraged participants to send weekly video messages via tablets. Second, the physical exercise sessions at home were led by an exercise professional through ZOOM to enhance the adherence and effectiveness of exercise. Third, the cognitive training application was designed to allow the study staff to access all participants’ data via an administrative homepage, thereby allowing them to monitor and enhance adherence to cognitive training at home. Additionally, we conducted online cognitive training sessions focusing on homework via ZOOM to improve the adherence and effectiveness of cognitive training. Fourth, a fully non–face‐to‐face intervention is likely to result in decreased adherence among older adults. We therefore implemented a hybrid model that integrated face‐to‐face and remote programs to achieve high adherence. Fifth, adherence was improved by the close relationship between the dedicated study coordinator and the participants, as well as between participants within the same group.

This study also showed that MI was relatively safe among older adults with MCI. Intervention‐related AEs were reported in only four cases. These were musculoskeletal AEs. In one case, a rib fracture occurred during a face‐to‐face exercise session. Caution should be exercised when performing physical exercises in elderly individuals with poor flexibility and osteopenia.

In the intervention group, the RBANS total scale index score showed a significant improvement by week 12 compared to the control group. This result is consistent with a previous study indicating that cognitive function improved after 12 weeks of cognitive intervention in individuals with MCI,^45^ as well as with a study demonstrating cognitive improvements following 12 weeks of exercise intervention in at‐risk elderly population.^46^ Although a significant difference between the MI and control groups was observed at 12 weeks, the difference further increased at 24 weeks, supported by a significant interaction between visit and trial group. The improvement in the RBANS total scale index score for the control group may be due to a test‐related learning effect. Additionally, the controls, who agreed to participate in the study, may have been motivated to improve their lifestyle habits. As a result, they may have consulted the provided educational booklets on risk factors and lifestyle guidelines for dementia prevention, undertaking lifestyle improvements such as exercise and dietary control independently. This self‐initiated effort could have contributed to their improvement. However, the improvement in the control group was still below the minimum clinically important difference of 8 points on the RBANS total scale index score.^43^ The individual index scores for the memory, language, and attention domains showed greater numerical improvements in the MI group than in the control group, but these differences were not statistically significant. This may be due to insufficient study power to detect significance for each cognitive domain. However, the visuoconstruction index score significantly improved in the MI group compared to the control group. Previous studies in patients with early AD and in older adults at risk of dementia also found significant improvements in visuoconstruction index scores in the MI group compared to the control group.^15^, ^16^ Dynamic cognitive training using a computer program is likely effective for enhancing visuoconstruction abilities, as it provides stronger visual stimulation and incorporates three‐dimensional space. No differences were observed between the MI and control groups in changes to vascular risk factors. This may be because the control group, like the intervention group, also received drug treatments for these risk factors during the study period. In the FINGER trial, no significant differences were found in vascular risk factor changes between the intervention and control groups.^11^ This suggests that MI may act directly on the brain rather than affecting cognitive function through regulation of vascular risk factors.

The analysis of the MMSE and RBANS revealed some differences in the subgroup analyses. However, when greater emphasis was placed on the RBANS total scale index score, which was the primary outcome measure, MI appeared to be more effective in amnestic MCI than in nonamnestic MCI. These results are very encouraging, as amnestic MCI is more likely to be caused by MCI due to AD than nonamnestic MCI.^47^ Thus, MI may have a synergistic or additive effect in patients with MCI treated with lecanemab or donanemab. The MI group showed significant beneficial effects on cognition, as measured by the RBANS, in APOE ε4 noncarriers and carriers. Compared with the control group, MMSE scores were also significantly improved in APOE ε4 noncarriers, but not in APOE ε4 carriers. Therefore, MI appeared to be effective in both groups, with the effect seeming to be greater in APOE ε4 noncarriers. A recent study observed a trend favoring a lower increase in beta‐amyloid deposition on positron emission tomography (PET) among APOE ε4 noncarriers with MI.^48^ The results of our study are similar to those of the RCTs on lecanemab and donanemab, in that cognitive function improved in the intervention group compared to the control group in both APOE ε4 carriers and noncarriers, with the absolute degree of improvement being greater in APOE ε4 noncarriers.^5^, ^6^ MI showed significant beneficial effects on cognition in females. A trend favoring MI on cognition was noted in males. Significant results may not have been obtained due to an insufficient number of males to demonstrate the intervention effect.

About one‐third of participants needed additional assistance in using their tablet PCs at least once, and a third called for help connecting to ZOOM at least once. Approximately 10% required assistance with connecting to ZOOM for more than 3 weeks, and about 5% needed help using a tablet PC more than 10 times. However, most participants became accustomed to using tablet PCs and accessing ZOOM within 1‐2 weeks. The average education level of the participants was not high. Further, most participants had never used a tablet PC or ZOOM platform before participating in the study. Additionally, because the participants had MCI, they may have required more assistance. Nonetheless, these results indicate that MI using a video communication platform and digital applications is feasible in MCI with sufficient early education.

This study had several limitations. First, only single‐blind design was applied. Further, there is a possibility that full blinding was not maintained. In addition, the study coordinators who assessed adherence were aware of the participants in the intervention group. Second, the control group knew that MI was available after the study was concluded. This awareness may have partially contributed to the decline in cognitive function and depressive symptoms observed in the control group. Third, MCI was clinically diagnosed without biomarkers. Differences in the proportion of patients with progressive or nonprogressive MCI between the MI and control groups may have affected the results. Fourth, the intervention period of this study was relatively short compared to other large multidomain RCTs such as FINGER.^11^, ^12^, ^13^ Long‐term follow‐up studies are needed to confirm the effects of MI in preventing dementia and delaying the progression of MCI or AD. Fifth, to ensure diversity and equity, hospitals across the country participated in the study, with six hospitals receiving referrals from affiliated public health centers. Patients referred from public health centers are often of lower economic status. However, future studies that include subjects from more diverse regions, various ethnicities, and different socioeconomic levels are needed. Sixth, this study did not demonstrate the effects of educational level and digital literacy on MI. While MI showed promise, further research is needed to fully address these factors and their impact on MI.

In conclusion, this trial demonstrated that MI exerts significant beneficial effects on cognition in patients with MCI. MI showed significant beneficial effects on cognition in APOE ε4 carriers and noncarriers. MI seems to be more effective in patients with amnestic MCI than in those with nonamnestic MCI. Further research is required to strengthen generalizability and to evaluate the effectiveness of customized programs based on the etiology of MCI.

## CONFLICT OF INTEREST STATEMENT

Jee Hyang Jeong and Seong Hye Choi consult for PeopleBio Co. Ltd. So Young Moon, Chang Hyung Hong, Jee Hyang Jeong, Yoo Kyoung Park, Hae Ri Na, and Seong Hye Choi are shareholders of Rowan Inc. Jiwoo Jung reported being an employee of Rowan Inc. The remaining authors report no conflicts of Interest. Author disclosures are available in the Supporting Information.

## CONSENT STATEMENT

All of the participants provided written informed consent to participate in the study.

## Supporting information



Supporting information

Supporting information
