# Supplementary material for: South Korean study to prevent cognitive impairment and protect brain health through multidomain interventions via face‐to‐face and video communication platforms in mild cognitive impairment (SUPERBRAIN‐MEET): A randomized controlled trial
Source: Alzheimers Dement. 2025 Jan 22;21(2):e14517. doi: 10.1002/alz.14517 (PMC11848216; doi:10.1002/alz.14517)
Supplement: Supplementary file 1 — Supporting information [file ALZ-21-e14517-s001.docx]

**Supplementary Materials**

Table S1. Multidomain intervention

| Intervention component | Content |
| --- | --- |
| Overseeing and managing metabolic and vascular risk factors | Prior to the intervention, metabolic and vascular risk factors were assessed through blood tests, alcohol and smoking habits, blood pressure, weight, body mass index, and waist circumference. At baseline and week 12, participants met with a study physician, who informed them about their risk factors and prescribe medications as necessary. At baseline, participants were educated by a study nurse about risk factors and lifestyle guidelines for dementia prevention using educational materials loaded on a tablet PC. Patients met with the study nurse every 4 weeks to undergo measurement of anthropometric factors and to monitor smoking and alcohol consumption. If the participants’ risk factors did not improve, the study nurse re-educated the participants at week 12 using educational materials on the tablet PC. |
| Cognitive training | Cognitive training, performed using the tablet PC SUPERBRAIN application, targeted the cognitive domains of episodic memory, executive function, attention, working memory, calculation, and visuospatial functions. During the first 8 weeks of the trial, participants underwent weekly group cognitive training sessions (lasting 50 min) led by a qualified healthcare professional, such as a psychologist, occupational therapist, or study nurse at a facility. They further participated in weekly self-administered cognitive training sessions at home lasting 30–40 min. Participants also performed weekly online cognitive training sessions focused on homework led by a qualified healthcare professional via the ZOOM platform. For the remaining 16 weeks of the trial, participants attended group cognitive training sessions led by a qualified health professional at the facility once every 2 weeks. During weeks that included group sessions, participants also attended weekly self-administered cognitive training sessions at home. Further, participants attended weekly online cognitive training sessions focused on homework led by qualified healthcare professionals via the ZOOM platform. During weeks that did not include group sessions, participants attended self-administered cognitive training sessions twice weekly at home. They further attended online cognitive training sessions focused on homework led by qualified healthcare professionals twice weekly via the ZOOM platform. |
| Physical exercise | The physical exercise program comprised aerobic exercise, balance-improving exercises, flexibility-enhancing activities, strength-building exercises targeting major muscle groups, and movements involving the fingers and toes. Portable equipment such as elastic bands, nine numbered floor plates, and chairs were utilized. Exercise sessions covering a variety of exercises were conducted thrice per week, with each session lasting 50 min. Qualified exercise professionals led the exercise program both at a facility and via the ZOOM platform at home. The intensity of the exercise was increased, and the content of the exercise was modified every 8 weeks. During the first 8 weeks of the trial, participants attended weekly group exercise sessions, led by a qualified exercise professional at the facility. They further participated in online exercise sessions twice a week at home via the ZOOM platform. During the remaining 16 weeks of the trial, participants attended group exercise sessions at the facility every 2 weeks. During weeks with group sessions, participants participated in online exercise sessions performed twice a week at home via the ZOOM platform. During weeks without group sessions, participants participated in online exercise sessions three times a week at home using the ZOOM platform. |
| Nutritional guidance | Nutritional intervention was performed over three individual telephone consultations (30 min each) with a study dietitian, with additional viewing of nutrition education videos created by a nutrition professor on a tablet PC during a biweekly facility visit. The individual telephone consultations were performed at weeks 2, 4, and 8 and were tailored to the participant’s daily dietary needs and included education on customized diets to manage individual vascular risk factors. Nutrition education videos provided dietary education, hands-on exercises to promote dietary changes, and advice on how to prepare meals with recommended ingredients through cooking classes. As recommended by the Mediterranean-Dietary Approaches to Stop Hypertension diet Intervention for Neurodegenerative Delay (MIND) diet, participants were advised to consume at least 3 servings of whole grains per day; six or more servings of dark green vegetables per week; one or more serving of other vegetables per day; five or more servings of nuts per week; four or more servings of beans or legumes per week; berries and poultry at least twice per week; and fish at least once per week. They were further recommended to use olive oil as cooking oil and to limit themselves to no more than a glass of wine per day for alcohol drinkers; cheese, fried food, and fast food less than once a week; less than five servings per week of pastries and sweets; and less than four servings per week of red meat and associated products. Participants were motivated by completing the MIND diet checklist each week using a tablet PC. |
| Motivational enhancement | Motivational enhancement was performed four group counseling sessions, each lasting 50 min, led by a study coordinator at weeks 1, 2, 12, and 24. The motivational enhancement program aimed to instigate, sustain, and reinforce motivation, thereby serving as a psychological resource to support the continuity of dementia prevention activities. Participants’ motivation and self-efficacy levels were assessed during each of the motivational enhancement sessions. Moreover, the family coach program allowed a family member to reinforce a participant’s motivation. Participants received video messages from their families or research staff cheering them on, and were provided with opportunities to perform self-assessment of their achievements in the form of pop-up notifications every week before the tablet-based cognitive intervention. |

Table S2. Results of RBANS total scale index score sensitivity analyses

| Type of sensitivity analysis | Adjusted mean change from baseline to 24 weeks (SE) | |  | Adjusted mean between-group difference in 24-week change from baseline (95% CI) | |
| --- | --- | --- | --- | --- | --- |
|  | MI (n=142) | Control (n=135) |  | MI vs. Control | *P*^*^ |
| MMRM repeated on mITT subjects with missing data imputed via  multiple imputation  approach | 8.44 (0.66) | 4.24 (0.65) |  | 4.19 (1.91–6.48) | < 0.001 |

MI, multidomain intervention; RBANS, Repeatable Battery for the Assessment of Neuropsychological Status; MMRM, mixed model for repeated measures; mITT, modified intention-to-treat. ^*^Linear mixed model that included subject and study center as random effects and the baseline score as a covariate, with trial group, visit, center by recruitment rate (≥2 subjects per month or <2 subjects per month), and trial group × visit interaction as fixed effects.

Table S3. Adjusted mean differences in the change in each index score of the RBANS from baseline to 24 weeks between the multidomain intervention and control groups (per-protocol population)

|  | Adjusted mean change from baseline to 24 weeks (SE) | |  | Adjusted mean between-group difference in 24-week change from baseline (95% CI) | |
| --- | --- | --- | --- | --- | --- |
|  | MI (n=130) | Control (n=123) |  | MI vs. Control | *P*^*^ |
| Primary outcome |  |  |  |  |  |
| Total scale | 8.27 (0.63) | 4.05 (0.65) |  | 4.22 (1.92–6.52) | < 0.001 |
| Secondary outcomes |  |  |  |  |  |
| Immediate memory | 7.07 (0.69) | 5.16 (0.72) |  | 1.91 (-0.69–4.51) | 0.150 |
| Visuoconstruction | 4.56 (0.88) | -0.71 (0.91) |  | 5.24 (1.96–8.53) | 0.002 |
| Language | 4.75 (0.81) | 2.11 (0.83) |  | 2.65 (-0.33–5.62) | 0.081 |
| Attention | 1.49 (0.63) | 0.29 (0.66) |  | 1.20 (-1.13–3.53) | 0.312 |
| Delayed memory | 8.52 (0.86) | 6.14 (0.89) |  | 2.38 (-0.79–5.54) | 0.141 |

MI, multidomain intervention; RBANS, Repeatable Battery for the Assessment of Neuropsychological Status. *Linear mixed model that included subject and study center as random effects and the baseline score as a covariate, with trial group, visit, center by recruitment rate (≥2 subjects per month or <2 subjects per month), and trial group × visit interaction as fixed effects.

Table S4. Mean changes in the secondary outcome measures from baseline to study end in participants receiving multidomain intervention and controls (per-protocol population)

|  | Baseline scores | |  | Changes from baseline to study end | |  |  |
| --- | --- | --- | --- | --- | --- | --- | --- |
|  | MI  (n=130) | Control (n=123) |  | MI  (n=130) | Control (n=123) |  | *P* |
| Mini-Mental State Examination^‡^ | 26.5 (2.1) | 26.5 (2.5) |  | 0.3 (2.0) | -0.7 (2.4) |  | <0.001^*^ |
| CDR-SB | 1.13 (0.84) | 1.37 (0.91) |  | -0.03 (0.50) | 0.06 (0.64) |  | 0.154^†^ |
| PRMQ | 34.4 (10.7) | 36.4 (11.3) |  | -1.6 (9.8) | -1.2 (9.7) |  | 0.237^†^ |
| PRMQ by caregiver | 35.7 (10.7) | 36.5 (11.8) |  | -1.1 (9.2) | 0.6 (9.8) |  | 0.090^*^ |
| Geriatric Depression Scale-15 items | 4.4 (3.8) | 5.0 (3.9) |  | -1.3 (3.1) | -0.7 (3.2) |  | 0.014^*^ |
| Bayer Activities of Daily Living | 2.24 (1.11) | 2.45 (1.30) |  | 0.03 (1.22) | 0.06 (1.32) |  | 0.673^†^ |
| QOL-AD^‡^ | 33.4 (4.0) | 32.2 (5.0) |  | 2.0 (4.4) | 0.9 (3.7) |  | 0.003^*^ |
| Pittsburgh Sleep Quality Index | 6.2 (4.1) | 6.7 (4.0) |  | -0.3 (3.1) | -0.2 (3.2) |  | 0.375^*^ |
| Short Physical Performance Battery^‡^ | 10.1 (1.8) | 9.9 (2.0) |  | 0.7 (1.8) | 0.2 (1.5) |  | 0.001^*^ |
| 30 s sit-to stand test^‡^ | 13.3 (3.7) | 14.0 (4.8) |  | 3.4 (5.1) | 0.8 (4.2) |  | <0.001^†^ |
| 2 min stepping test^‡^ | 97.5 (29.8) | 96.6 (34.6) |  | 27.0 (29.0) | 2.3 (30.7) |  | <0.001^†^ |
| Nutrition Quotient for Elderly^‡^ | 67.5 (9.8) | 64.4 (10.5) |  | 3.7 (6.8) | 0.6 (7.8) |  | <0.001^*^ |
| Mini Nutritional Assessment^‡^ | 12.2 (1.9) | 11.8 (2.1) |  | 0.2 (1.7) | 0.3 (2.2) |  | 0.778^†^ |
| Systolic BP, mmHg | 126.1 (12.7) | 127.8 (13.5) |  | 0.8 (14.3) | 2.0 (15.3) |  | 0.203^*^ |
| Diastolic BP, mmHg | 70.5 (10.4) | 70.3 (9.0) |  | -0.2 (11.0) | 0.2 (10.8) |  | 0.840^*^ |
| Body Mass Index, kg/m^2^ | 23.7 (3.0) | 23.9 (3.6) |  | -0.12 (0.83) | -0.01 (1.17) |  | 0.868^†^ |
| HbA1c, % | 6.0 (0.6) | 5.9 (0.6) |  | -0.06 (0.30) | -0.04 (0.35) |  | 0.509^†^ |
| Fasting glucose, mg/dL | 111.6 (34.7) | 106.8 (23.0) |  | -2.1 (37.0) | -0.7 (28.2) |  | 0.874^†^ |
| Total cholesterol, mg/dL | 173.6 (37.6) | 187.0 (44.7) |  | -9.8 (29.4) | -14.9 (41.6) |  | 0.849^†^ |
| LDL-cholesterol, mg/dL | 95.9 (32.8) | 105.5 (37.8) |  | -6.8 (26.0) | -10.9 (35.6) |  | 0.800^†^ |
| HDL-cholesterol, mg/dL | 56.4 (15.0) | 57.1 (13.4) |  | 1.2 (9.1) | 0.1 (8.7) |  | 0.354^*^ |
| Triglyceride, mg/dL | 118.8 (71.7) | 140.0 (89.0) |  | -6.8 (65.7) | -13.5 (84.0) |  | 0.813^†^ |
| Self Determination Index^‡^ | 16.8 (19.6) | 10.9 (19.8) |  | 10.0 (19.9) | -1.0 (18.0) |  | <0.001^*^ |

Values are presented as mean (SD). MI, multidomain intervention; CDR-SB, Clinical Dementia Rating Scale-Sum of Boxes; PRMQ, Prospective Retrospective Memory Questionnaire; QOL-AD, Quality of Life in Alzheimer’s disease; BP, blood pressure; HbA1c, Hemoglobin A1c; LDL, low-density lipoprotein; HDL, high-density lipoprotein. ^*^Analysis of covariance with the baseline score as a covariate. ^†^Nonparametric partial correlation adjusted for the baseline score between treatment group and change in each outcome from baseline to study end. ^‡^Higher scores indicate better performance.
